# Immunodominant Cytomegalovirus Epitopes Suppress Subdominant Epitopes in the Generation of High-Avidity CD8 T Cells

**DOI:** 10.3390/pathogens10080956

**Published:** 2021-07-29

**Authors:** Kirsten Freitag, Sara Hamdan, Matthias J. Reddehase, Rafaela Holtappels

**Affiliations:** Institute for Virology and Research Center for Immunotherapy (FZI), University Medical Center of the Johannes Gutenberg-University Mainz, 55131 Mainz, Germany; kfreitag@uni-mainz.de (K.F.); sara.hamdan@uni-mainz.de (S.H.); matthias.reddehase@uni-mainz.de (M.J.R.)

**Keywords:** antigen presentation, antigenic peptides, CD8 T cells, cytomegalovirus, epitope(s), immunodominance, immunotherapy, protective immunity, vaccine design

## Abstract

CD8^+^ T-cell responses to pathogens are directed against infected cells that present pathogen-encoded peptides on MHC class-I molecules. Although natural responses are polyclonal, the spectrum of peptides that qualify for epitopes is remarkably small even for pathogens with high coding capacity. Among those few that are successful at all, a hierarchy exists in the magnitude of the response that they elicit in terms of numbers of CD8^+^ T cells generated. This led to a classification into immunodominant and non-immunodominant or subordinate epitopes, IDEs and non-IDEs, respectively. IDEs are favored in the design of vaccines and are chosen for CD8^+^ T-cell immunotherapy. Using murine cytomegalovirus as a model, we provide evidence to conclude that epitope hierarchy reflects competition on the level of antigen recognition. Notably, high-avidity cells specific for non-IDEs were found to expand only when IDEs were deleted. This may be a host’s back-up strategy to avoid viral immune escape through antigenic drift caused by IDE mutations. Importantly, our results are relevant for the design of vaccines based on cytomegaloviruses as vectors to generate high-avidity CD8^+^ T-cell memory specific for unrelated pathogens or tumors. We propose the deletion of vector-encoded IDEs to avoid the suppression of epitopes of the vaccine target.

## 1. Introduction

The establishment of latent infection after the resolution of productive primary infection in the otherwise healthy, immunocompetent host is a hallmark of all members of the herpesvirus family. According to the definition by Roizman and Sears, which was originally proposed for the α-herpesvirus subfamily but then extended also to the members of the β- and γ-herpesvirus subfamilies, latent infection, briefly referred to as “latency”, is characterized by lifelong maintenance of non-replicating but replication-competent viral genomes in absence of infectious virus [1]. In addition to acute disease caused after primary infection by uncontrolled virus spread and associated histopathology in an immunologically immature or immunodeficient host, medical importance results from the risk of productive reactivation of latent virus. This can result in recrudescent organ disease in patients immunocompromised due to immunosenescence or by therapies of unrelated diseases that involve immunosuppression.

These principles apply also to the pathobiology of human cytomegalovirus (hCMV), which is the prototype member of the herpesvirus β-subfamily, as well as to all known primate and rodent cytomegaloviruses (CMVs), including murine cytomegalovirus (mCMV) [2,3]. Congenital primary hCMV infection of the embryo/fetus after diaplacental transmission is the most frequent viral cause of birth defects, which is historically known as cytomegalic inclusion disease [4,5,6]. Furthermore, hCMV reactivation associated with immunocompromising conditions in transplantation settings remains a challenging problem at all transplantation centers worldwide, despite the availability of antiviral drugs. Specifically, transient immunodeficiency in the reconstitution phase after the therapy of hematopoietic malignancies by hematoablative treatment followed by hematopoietic (stem) cell transplantation (HCT) opens a “window of opportunity” for latent hCMV to reactivate in myeloid lineage cells of the donor hematopoietic transplant ([7,8,9,10,11,12,13], for clinical overviews, see [14,15]) as well as in latently infected tissue cells of the recipient (for an overview and discussion, see [16]), often resulting in a life-threatening interstitial pneumonia [15,17]. A particularly high risk of hCMV reactivation and disease results from cytokine stimulation by graft-versus-host (GvH) reaction as such [11], as well as from iatrogenic immunosuppression to prevent GvH disease (GvHD) in histoincompatible, “allogeneic” HCT with HLA or minor histocompatibility antigen disparities between graft and recipient [18,19], as well as to prevent graft rejection by host-versus-graft (HvG) reaction in recipients of allogeneic solid organ transplantation (SOT) (for clinical overviews, see [20,21]).

The host-species specificity of CMVs limits the clinical investigation of the hCMV pathomechanisms and restricts experimental studies of hCMV pathogenesis, therapy, and immune response to immunologically humanized mouse models, including those with human tissue implants [22,23,24,25,26], to aspects that do not depend on a homologous cytokine network cross-talk or on organ functionality (discussed in [27]). Despite all incontestable differences in both virus and host genetics, the mouse model employing mCMV for a natural virus–host pair has proven its predictive value for identifying generally valid principles of CMV pathogenesis and immune control in various experimental settings, including infection after HCT [27,28]. As a paradigm, immunotherapy of CMV disease in the immunocompromised host by adoptive transfer of virus-specific CD8^+^ T cells was originally developed in the mouse model ([29,30], reviewed in [27,31]) years before it went into first clinical trials [32,33]. Just recently, mouse models explained lethal CMV disease after allogeneic HCT by a histoincompatibility-associated failure in the reconstitution of high-avidity virus-specific CD8^+^ T cells [34,35].

The development of a vaccine that induces humoral as well as cellular immunity is a priority goal for preventing congenital CMV infections [4,36,37,38]. In HCT, immunotherapy by adoptive transfer of antiviral CD8^+^ T cells for bridging the vulnerable phase of transient immunodeficieny between hematoablative leukemia/lymphoma therapy and lympho-hematopoietic reconstitution has become the last therapeutic option against CMV disease caused by the reactivation of drug-refractory virus variants [39,40,41,42,43,44,45]. An optimal choice of the viral antigens to be expressed by a vaccine or to be targeted by adoptively transferred antiviral CD8^+^ T cells is a central issue for vaccine and immunotherapy design, respectively. As a logical rationale, the first choice was the inclusion of antigens that are known to elicit strong immune responses in natural infections. Most studies and clinical trials on cytoimmunotherapy were performed with CD8^+^ T cells specific for the hCMV tegument protein UL83/pp65, because it was known to induce high frequencies of cytolytic CD8^+^ T lymphocytes (CTL) in natural infections [46,47,48,49], although the unusual expansion of these cells [49] may actually have indicated a low protective efficacy in preventing hCMV reactivation from latency. From a present-day perspective, such cells were stimulated to extensive proliferation by antigen expressed after virus reactivation, but they may have failed to stop reactivation because of a too low average avidity of the involved T-cell receptors (TCRs) for the presented peptide-MHC/HLA class-I complexes. These considerations raise the question and concern of whether just reproducing an insufficient natural immune response by vaccination or cellular immunotherapy is really a good decision. The selection of high-avidity cells for adoptive transfer [31,50,51,52] and transduction of cells with high-avidity TCRs [53,54] are practicable options for protective CD8^+^ T-cell immunotherapy, whereas, as far as we can judge, designing a vaccine that preferentially activates high-avidity responses will be more demanding.

In a comprehensive approach to define the human T-cell immunome of hCMV, Sylwester et al. [55] arrived at the conclusion that the memory T-cell response is broadly targeted against viral peptides derived from many open reading frames (ORFs). Actually, 107 out of 213 ORFs were found to contribute one or more epitopes recognized by CD8^+^ T cells. However, such a broad repertoire of epitopes applies only on the population level and is determined by MHC/HLA polymorphism. In fact, some of the non-antigenic ORFs in that study might have reflected MHC/HLA genotypes not covered by the study cohort. On the level of individuals, the repertoire of used epitopes is narrowed by the individual heterozygotic set of MHC/HLA class-I genes. Specifically, analysis of CMV-experienced, “seropositive” individuals revealed a median number of 8 ORFs that contributed epitopes recognized by CD8^+^ T cells, with a range from 1 to 31 ORFs [55]. Considering that inbred strains of mice are genetically “individual-like” but with a homozygotic set of MHC class-I genes, antigenic ORF usage reported for mCMV-primed BALB/c (*H-2^d^*) (reviewed in [31,56]) and C57BL/6 (*H-2^b^*) mice [57] lies within this range reported for human individuals. Among those few antigenic ORFs, a hierarchy exists in terms of magnitude of the CD8^+^ T-cell response, operationally classifying epitopes as immunodominant and non-immunodominant or subordinate epitopes, IDE and non-IDE, respectively.

As an extension of our own previous studies on the relative importance of IDEs and non-IDEs for antiviral protection in different immunotherapy settings [58,59,60], we address here the question of whether hierarchy in the CD8^+^ T-cell response to epitopes is based on intrinsic differences, for instance the size of the naïve precursor pool, or if hierarchy reflects competition at the level of antigen presentation and recognition. Notably, our data provide evidence to conclude that the CD8^+^ T-cell response to non-IDEs profits from a deletion of IDEs not primarily by an increase in pool size, that is in total numbers of progeny, but rather by a preferential expansion of the pool of antivirally protective cells with high functional avidity. This finding is of importance for the design of CMV-vector vaccines [61,62,63,64,65], as IDEs not removed from the vector might not only suppress the high-avidity response to non-IDEs of the vector itself but also the high-avidity response to epitopes of the vaccine target pathogen or tumor. In the extreme, by such a mechanism, target epitopes chosen for inclusion in the vaccine because of being IDEs might even be turned into non-IDEs when expressed in the genetic context of the recombinant vaccine virus.

## 2. Materials and Methods

### 2.1. Mice, Viruses, and Infection Procedures

Female BALB/cJ (haplotype *H-2^d^*) mice were bred and housed under specified-pathogen-free (SPF) conditions by the Translational Animal Research Center (TARC) at the University Medical Center of the Johannes Gutenberg-University Mainz.

Immunocompetent mice were used in experiments as young adults at an age of 8 to 12 weeks. Local intraplantar infection was performed by injection of 10^5^ plaque-forming units (PFU) of cell culture-propagated, purified virus into the left hind footpad. For the different experiments, we used bacterial-artificial chromosome (BAC)-derived mCMV-BAC^W^ [66] or recombinant mCMVs lacking or expressing IDEs (antigenic peptides IE1/m123, m164, M105, and m145) due to C-terminal point mutations X9A or back-mutations A9X, which are here briefly referred to as ΔIDE [59] and rev-IDE [67], respectively. Latent infection was routinely confirmed by the presence of viral genomes in tissues in the absence of infectious virus [65,68].

### 2.2. Preparation of Single-Cell Suspensions from Lungs and Spleen

Mice were lethally anesthetized by carbon dioxide inhalation, and mononuclear leucocytes from lung tissue were isolated essentially as described previously ([65], and references therein). In brief, lungs were perfused via the right ventricle to remove circulating cells from the capillary bed of the lungs. Lungs were excised, tracheae, bronchi, and pulmonary lymph nodes were discarded, and the lung lobes were minced. The digestion of tissue collected from 4–5 lungs was performed in 15 mL of supplemented DMEM, containing collagenase A (1.6 mg/mL, catalog No. 10 103 586 001; Roche, Mannheim, Germany) and DNase I (50µg/mL, catalog No. DN-25; Sigma-Merck, Darmstadt, Germany) for 1 h at 37 °C with constant stirring. Mononuclear leucocytes were enriched by density-gradient centrifugation for 30 min at 760× *g* on lymphocyte separation medium Histopaque-1077 (catalog No. 10771, Sigma-Merck). For preparing single-cell suspensions of splenocytes, spleens were minced and passed through a cell strainer, followed by the lysis of erythrocytes.

### 2.3. Peptides and Quantitation of Functional Epitope-Specific CD8^+^ T Cells

Epitopes corresponding to reported antigenic peptides presented by MHC class-I molecules K^d^, D^d^, and L^d^ are derived from the mCMV open reading frames (ORFs) m04, m18, M45, M83, M84, M105, m123/IE1, m145, and m164 (listed in [31,56]; for an update see [60]). Custom peptide synthesis with a purity of >80% was performed by JPT Peptide Technologies (Berlin, Germany).

At indicated times after intraplantar infection, CD8^+^ T cells were immunomagnetically purified from spleen cell suspensions (pool of 5–10 mice per time of analysis) to serve as responder cells in an IFNγ-based enzyme-linked immunospot (ELISpot) assay ([65,69], and references therein). For quantitating functional, epitope-specific CD8^+^ T cells, synthetic peptides were exogenously loaded at the indicated molar concentrations on P815 (*H-2^d^*) mastocytoma cells for serving as stimulator cells in the assay. In brief, graded numbers of CD8^+^ T cells were seeded with the peptide-loaded stimulator cells in triplicate microcultures. After 18 hrs of incubation, spots, each representing a specifically sensitized IFNγ-secreting cell, were counted automatically, based on standardized criteria using ImmunoSpot S4 Pro Analyzer (Cellular Technology Limited, Cleveland, OH, USA).

### 2.4. Cytofluorometric Analyses

Single-cell suspensions were prepared from spleen and lungs as described above. Unspecific staining was blocked with unconjugated anti-FcγRII/III antibody (anti-CD16/CD32, clone 93; eBioscience, San Diego, CA, USA), and cells were specifically stained with the following antibodies for multi-color cytofluorometric analyses: ECD-conjugated anti-CD8α (clone 53–6.7; Beckman Coulter, Krefeld, Germany), FITC-conjugated anti-KLRG1 (clone 2F1, eBioscience), PE-Cy5-conjugated anti-CD127 (clone A7R34, eBioscience), and PE-Cy7-conjugated anti-CD62L (clone MEL-14, Beckman Coulter). Epitope-specific cells were identified by staining with PE-conjugated, peptide-folded MHC-I dextramers H-2Ld/YPHFMPTNL (m123/IE1), H-2Dd/AGPPRYSRI (m164), and H-2Dd/SGPSRGRII (m18) (Immudex, Copenhagen, Denmark). A lymphocyte live gate was routinely set in the forward vs. sideward scatter plot. Cytofluorometric analyses were performed with flow cytometer FC500 and CXP analysis software (Beckman Coulter).

### 2.5. Statistics and Determination of Avidity Distributions

For longitudinal immune response analyses, groups of age-matched mice were randomized before treatment, and data for the indicated read-out times after infection represent the experimental average for pooled samples (*n* = 5–10). Frequencies (most probable numbers, MPN) of cells responding in the ELISpot assay and the corresponding 95% confidence intervals were calculated by intercept-free linear regression analysis from the linear portions of regression lines based on spot counts from triplicate assay cultures for each of the graded cell numbers seeded [69] (Graph Pad Prism 6.04, Graph Pad Software, San Diego, CA, USA). Cumulative avidity plots show the measured frequencies of cells responding to the indicated peptide concentration and all lower concentrations. Based on MPN and the corresponding upper and lower 95% confidence limits, half-maximal effective concentration (EC_50_) values, representing peptide concentrations that result in half-maximal response of the cell population, were calculated with *Quest Graph™* EC_50_ Calculator (AAT Bioquest, Inc.; retrieved from https://www.aatbio.com/tools/ec50-calculator, accessed on 16 July 2021). Gaussian-like avidity distributions reveal frequencies of cells with an avidity defined by the respective peptide concentration indicated. These are deduced from the cumulative avidity distribution values by plotting the response increments between a peptide concentration and the next higher peptide concentration [65].

## 3. Results

### 3.1. Long-Term Kinetics of CD8^+^ T-Cell Memory during Latent Infection after Local Priming

It is a hallmark of all herpesviruses that, after resolution of productive primary infection, the viral genome is maintained in certain cell types in a non-replicative but reactivation-competent stage [1]. In the specific case of mCMV, latent viral genomes localize to endothelial cell (EC) types, as shown so far for liver-sinusoidal EC (LSEC) (reviewed in [70]) and for EC of the capillary bed in the lungs [68], with more EC types and their tissue localizations awaiting experimental verification (discussed in [16]). Notably, as it was discovered in the murine model, latency is linked to an immunological hallmark of CMV infection [70], namely an unusual kinetics of the CD8^+^ T-cell response that is characterized by a phenomenon known as “memory inflation” (MI) (for reviews, see [71,72,73,74]). In essence, after expansion of virus-specific CD8^+^ T cells constituting the primary immune response driven by antigens presented during acute infection, the pool size contracts in parallel to the resolution of productive infection, as it is the case also in non-chronic infections. However, after establishment of the non-productive latent infection, pool sizes of cells specific for a set of viral epitopes almost steadily expand over time. The expanding pool is made up by “inflationary” T effector-memory cells (iTEM), which are characterized by the cell surface marker phenotype KLRG1^+^CD127 (IL7R)^−^CD62L^-^ [68,75,76], which distinguishes them from KLRG1^+^CD127^+^CD62L^−^ double-positive effector cells (DPEC), KLRG1^−^CD127^−^CD62L^−^ early effector cells (EEC), KLRG1^−^CD127^+^CD62L^−^ conventional T effector-memory cells (cTEM), as well as from KLRG1^−^CD127^+^CD62L^+^ T central memory cells (TCM) [77]. As we have shown recently, the stochastic expression of viral genes and associated epitope presentation during mCMV latency drives MI made up by iTEM [68] and explains the immunological finding of stochastic expansions of individual epitope-specific iTEM clones [78]. This particular property raised substantial interest in promoting CMVs as vaccine vectors by replacing MI-driving epitopes of a CMV vector with foreign epitopes to generate a long-term and, moreover, self-amplifying memory against unrelated pathogens or against tumors (see the Introduction).

However, enthusiasm is somewhat dampened by a retrospective view on decades of clinical investigation of the human immune response to hCMV, arriving at the conclusion that MI is not regularly observed in human infection [79]. These, at first glance, conflicting data for mCMV and hCMV can be explained by the latent viral genome load that depends on the spread of virus during primary infection, which defines the probability of MI-driving viral gene expression during latency [68].

Based on this rationale, Adler and Reddehase [80] discussed the idea that congenital or neonatal hCMV infections, which are characterized by an extended period of productive primary infection, should lead to MI, whereas the prevention of viral spread by efficient early immune control prevents MI in people infected later in childhood or as adults. While the history of primary infection is a given and mostly unknown parameter in individuals, primary infection in a mouse model can be defined by the experimental conditions chosen. Thus, as we have shown recently, MI by iTEM can be induced by highly productive systemic infections, but not after local infection of immunocompetent mice in which productive infection is rapidly resolved [65].

**Figure 1:**
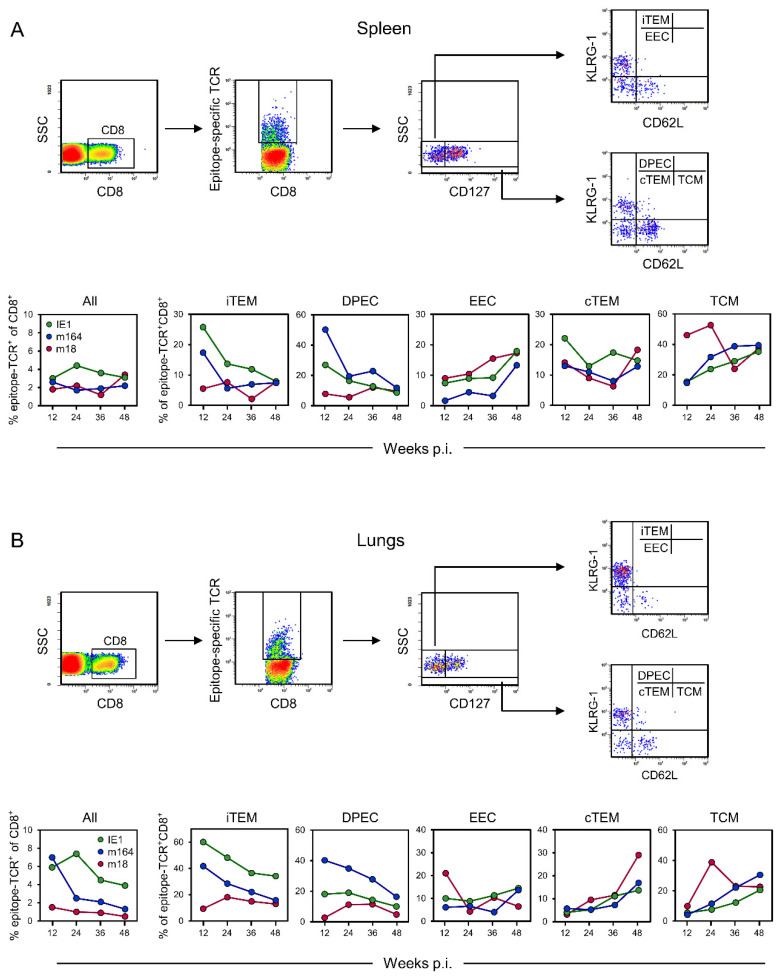
Cytofluorometric determination of viral epitope-specific CD8^+^ T-cell subpopulations in the time course after intraplantar infection of immunocompetent BALB/c mice with mCMV-BAC^W^. (**A**) Spleen, representing a lymphoid site. (**B**) Lungs, representing an extra-lymphoid site. (Top panels), gating strategy to define and quantitate viral epitope-specific subpopulations iTEM, DPEC, EEC, cTEM, and TCM. (Bottom panels), time course of the response to IDEs IE1 and m164, as well as non-IDE m18, separated into the subpopulations indicated. To average individual variation, cells isolated from 5–10 mice per time, depending on cell yield, were pooled for the analysis.

As local infection is the preferred route for application of a vaccine in humans, we extend here on our previous work in the BALB/c mouse model with intraplantar infection [65,69] to compare the long-term development of CD8^+^ T-cell memory specific for the two most prominent MI-driving IDEs m123/IE1 and m164 with that specific for not MI-driving non-IDE m18 (Figure 1). The spleen was chosen as a central lymphoid organ (Figure 1A), and the lungs were chosen as they represent a non-lymphoid organ and prominent tissue site of mCMV latency (Figure 1B).

At both sites, the most notable difference concerns the two KLRG1^+^ subpopulations iTEM and DPEC, which are known to be maintained only by continuous or at least the repetitive presence of antigen presentation [81]. While proportions of iTEM and DPEC were high in the beginning but declined over time for the two IDEs, which indicates a continuous decline in antigen presentation over time, they were low from the beginning and stayed low for non-IDE m18. This most likely reflects a generally infrequent stimulation by m18 peptide presentation and determines the classification of m18 as a non-IDE. For all three epitopes, we note an increase in EEC over time in the spleen but not in the lungs. We have no immediate explanation for this, also because there is generally little known about EEC. Contrary to EEC, cTEM accumulated over time in the lungs, in particular those specific for non-IDE m18, which can be explained by rapid loss of KLRG1 expression due to low and infrequent restimulation by m18-peptide presentation, so that only transiently generated iTEM and DPEC eventually feed the m18-specific cTEM pool. Mirror-inverted to iTEM and DPEC, proportions of TCM increased over time for both IDEs in spleen and lungs, which is consistent with a decline in antigen presentation and thus establishment of central memory. As a reminder for clarity, the frequencies of naïve precursor cells are too low for the quantitation by peptide-MHC multimer staining, and all detectable epitope-specific cells express CD44 [82], so that CD62L^+^ cells represent TCM. Notably, already early during latency, the proportion of m18-specific TCM was high in the spleen, which is consistent with infrequent restimulation from early on, whereas, at the same time, frequent restimulation by antigen presentation still kept IDE-specific cells in the iTEM and DPEC pools. In contrast to the situation in the spleen, the proportion of m18-specific TCM was found to be low at an early time in the lungs. This can be explained by the generation of TCM in the spleen, followed by an export to extra-lymphoid sites.

It is important to note that error statistics cannot be applied to T-cell responses during viral latency, because the immune response of mice differs by individual variation. The molecular basis for the individuality of the response kinetics is the recently discovered stochastic expression of viral genes during latency [68]. This finds its correspondence in stochastic stimulation and thus stochastic expansion or contraction of T-cell clones [78], which also determines the activation phenotypes in that only recently stimulated cells assume an iTEM phenotype. Accordingly, as shown previously in the related model of the immune response to mCMV after experimental HCT [60], independent response kinetics are never identical. Therefore, our data must be understood as an average response for each time of analysis.

### 3.2. Impact of IDEs on the Long-Term Kinetics of CD8^+^ T-Cell Memory Directed against Non-IDE m18

As the most salient difference in the response to IDEs IE1 and m164 compared to non-IDE m18, the data shown so far revealed higher proportions of the KLRG1^+^ subpopulations iTEM and DPEC, in particular at the earlier times in both spleen and lungs (Figure 1). As the expression of KLRG1 requires stimulation by antigen [81], this finding indicates a primarily low or non-sustained presentation of the m18 peptide or a higher avidity of IDE-specific cells that might successfully compete for space at immunological synapses where TCRs interact with peptide-MHC class-I complexes. Competition between CD8^+^ T cells at the level of antigenic peptide presentation has been described in a related mouse model of MI driven by mCMV [83], with fate decision made during acute infection found to program clonal dominance during MI ([84,85], for a review, see [86]). Therefore, we considered competition by IDE-specific cells as a possible mechanism explaining the low proportions of iTEM and DPEC specific for non-IDE m18. Previous demonstration of competition took the approach to suppress the response against intrinsic antigenic peptides of mCMV in C57BL/6 (haplotype *H-2^b^*) mice by expressing the unrelated “vaccine model epitope” SIINFEKL [83] under control of the particularly strong major IE promoter-enhancer of mCMV [87]. This left it open if competition was based on a more efficient epitope-encoding gene expression during latency [68], which leads to enhanced antigen synthesis, processing, and presentation. As alternatives, superior peptide affinity to the presenting MHC class-I molecule, to K^b^ in case of SIINFEKL, or a superior functional avidity of SIINFEKL-specific CD8^+^ T cells may have accounted for the observed difference.

**Figure 2:**
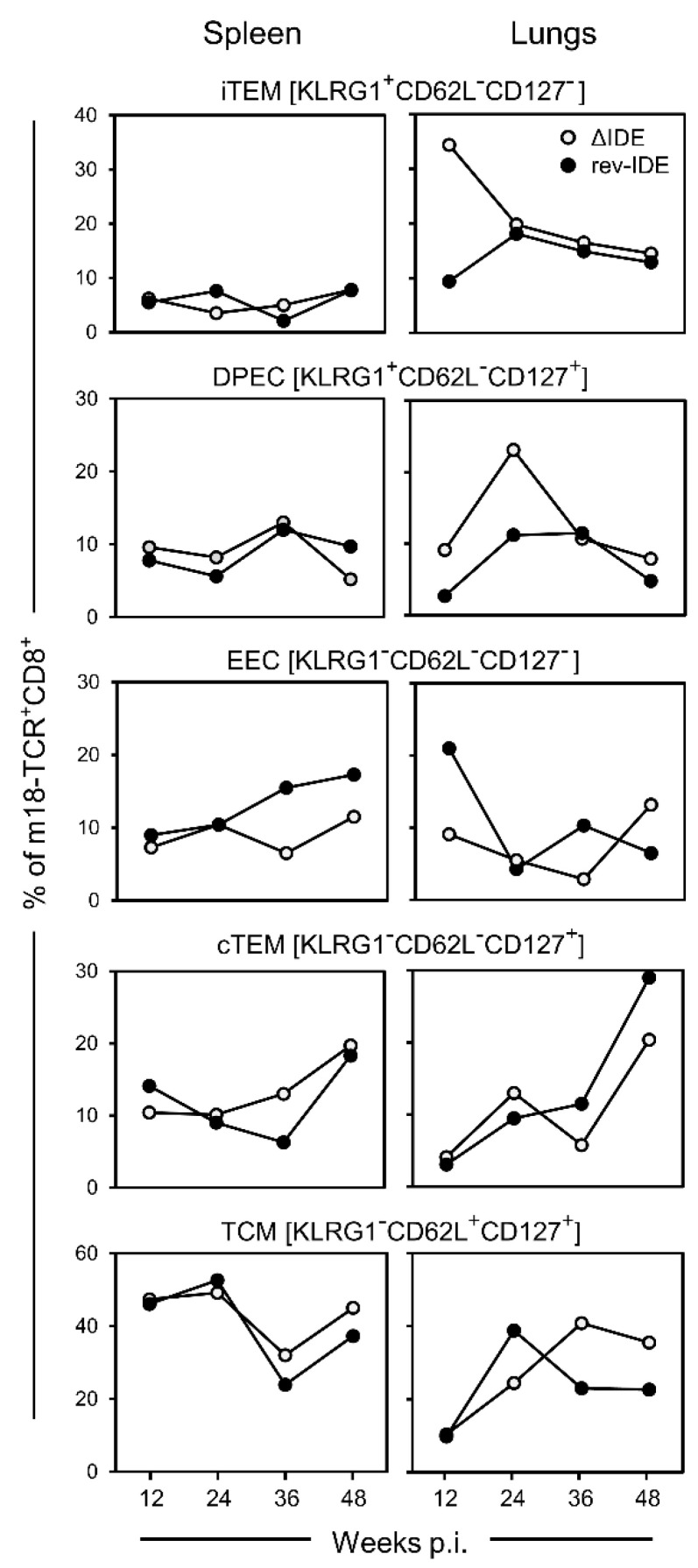
Impact of IDE antigenicity deletion on the long-term response to non-IDE m18 by CD8^+^ T-cell subpopulations iTEM, DPEC, EEC, cTEM, and TCM. Intraplantar infection was performed with recombinant mCMVs lacking or expressing functional IDEs, that is viruses ΔIDE and rev-IDE, respectively. To average individual variation, cells isolated from 5–10 mice per time, depending on cell yield, were pooled for the analysis.

Here, we have not introduced a “foreign” competitor peptide in a recombinant mCMV but, instead, addressed the modified though related question of whether mCMV intrinsic IDEs are actually the competitors for the mCMV-intrinsic non-IDEs. As an approach, we first compared the long-term kinetics of CD8^+^ T-cell memory in immunocompetent BALB/c mice in the absence and presence of IDEs after intraplantar infection with recombinant mCMV ΔIDE and its revertant mCMV rev-IDE, respectively (Figure 2).

As a more technical note, with the aim to minimize virus manipulation, we did not delete entire genes or complete peptide-coding sequences, but eliminated antigenicity and immunogenicity of IDEs very selectively by X9A point mutations of the respective C-terminal amino acid residues [88]. In addition to the elimination of IE1 and m164 antigenicity, we also mutated peptides M105 and m145 that in previous studies showed an inconsistent immunodominance profile and MI phenotype (reviewed in [56]).

At a glance, in terms of frequencies of responding cells of any of the subpopulations defined by activation marker phenotype, the response to non-IDE m18 in the spleen was not notably altered by functional deletion of the IDEs. In contrast, at least at earlier times during latency, KLRG1^+^ subsets iTEM and DPEC did clearly profit from the absence of IDEs in the lungs. As the expression of KLRG1 reflects recent activation by antigen, the deletion of IDEs has apparently released m18 from the inhibitory competition.

### 3.3. Functional Avidities of CD8^+^ T Cells Specific for IDEs

Staining with peptide-MHC class-I multimers detects CD8^+^ T cells that express epitope-specific TCRs, regardless of functional activity, and combination with the panel of cell surface markers indicates their activation stage. However, quantity alone is no warranty for functionality and protective activity in controlling the infection. It is decisive that the CD8^+^ T cells can recognize infected cells. As we have shown previously in our own group as well as in cooperation, the structural and functional avidity of the interaction between TCRs and peptide-MHC class-I complexes is critical for protective efficacy [31,52]. Moreover, as viral immune evasion proteins of CMVs interfere with cell surface trafficking of peptide-loaded MHC class-I complexes, and thus limit antigen presentation, only high-avidity CD8^+^ T cells can recognize infected cells to protect against virus spread ([65] and references on immune evasion therein). As it is difficult to directly quantitate endogenous antigen processing and presentation, we determined the concentration of synthetic antigenic peptide that is needed for exogenous loading of cell surface MHC class-I molecules to achieve a response equivalent to the response against infected target cells that present endogenously processed antigenic peptides under conditions of immune evasion. These studies revealed that the functional avidity of CD8^+^ T cells capable of detecting naturally processed and presented antigenic peptide must correspond to a loading concentration of ≤10^−9^ M of exogenously applied synthetic antigenic peptide [65].

**Figure 3:**
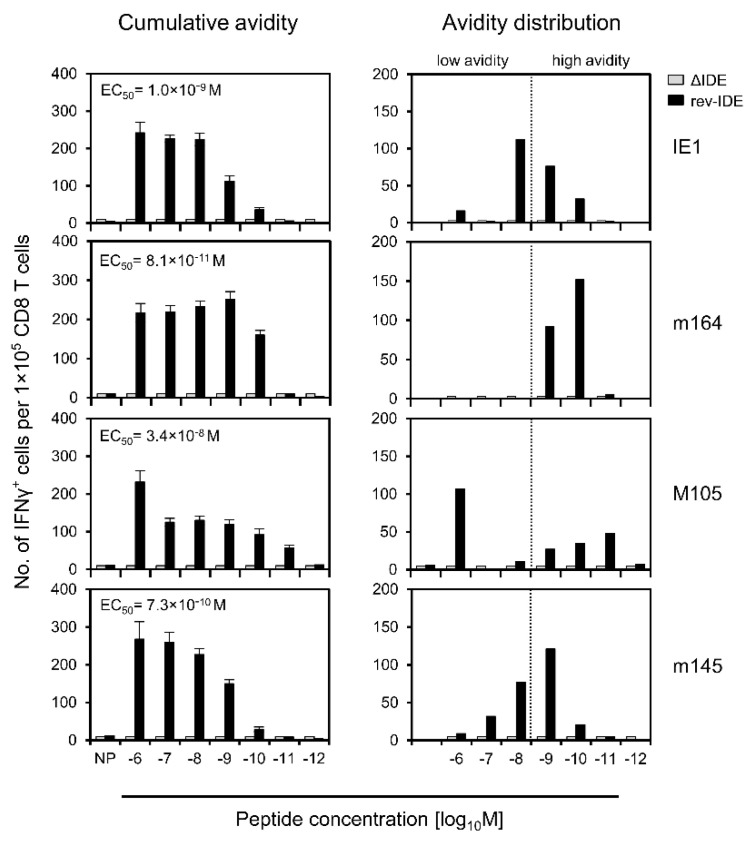
Detection and avidity distributions of epitope-specific IFNγ^+^CD8^+^ T cells. Immunocompetent BALB/c mice were infected via the intraplantar route with viruses ΔIDE (gray-shaded bars) or rev-IDE (black bars). At 40 weeks after infection, functional CD8^+^ T cells specific for the indicated four IDEs were quantitated based on IFNγ secretion in an ELISpot assay. Stimulation in the assay was achieved by P815 mastocytoma cells exogenously loaded with the respective synthetic peptides at the loading concentrations indicated. (Left column) Cumulative avidity plots. Bars represent the most probable numbers (MPN) of cells responding in the assay, as calculated by intercept-free linear regression. MPN values sum up all cells that respond to ≤ the test concentration indicated. Error bars represent the 95% confidence intervals. EC_50_ values were calculated from the MPN and the upper and lower confidence limit values. (Right column) Avidity distributions deduced from the cumulative avidities by plotting the response increments. The dotted line operationally separates cells with non-protective avidity, equivalent to >10^−9^ M peptide loading concentration (to the left), from cells with protective avidity, equivalent to ≤10^−9^ M peptide loading concentration (to the right). To average individual variation, cells isolated from 10 mice were pooled for the analysis.

Inspired by our grown awareness of the importance of recognition avidity [31,52,65], we first studied the functional avidities of IDE-specific IFNγ^+^ CD8^+^ T cells and verified the absence of IDE-specific responses after infection with mCMV ΔIDE as well as successful back-mutations A9X in mCMV rev-IDE (Figure 3). Only after infection with mCMV rev-IDE, cumulative avidity plots showed a response to the four IDEs with magnitudes depending on antigenic peptide loading concentrations, and EC_50_ values represent the average population avidities expressed as the peptide concentration that triggers the half-maximal response (Figure 3, left column). As an instructive example, an EC_50_ value of 3.4 × 10^−8^ M for IDE M105 indicated a functional avidity that is too low for the recognition of infected cells, and thus for protection. Yet, as EC_50_ values describe the average avidities of effector cell populations with a range of avidities of individual cells, the population can nevertheless comprise also cells of a protective avidity corresponding to the recognition of target cells loaded with ≤10^−9^ M of antigenic peptide. By plotting the response increments with increasing peptide concentrations, we determined the avidity distributions that precisely reveal the response magnitudes to each peptide concentration tested [65] (Figure 3, right column). Compared to EC_50_ values, this analysis has the advantage of showing if the effector cell population is unimodal with a more or less Gaussian-like avidity distribution, as it was here the case for peptides IE1, m164, and m145, or if it is heterogeneous in its composition representing different cell populations. Specifically, while the M105-specific response included many cells with a protective avidity of ≤10^−9^ M, the EC_50_ value was skewed to lower avidity by an unrelated population of very low-avidity cells that responded only to 10^−6^ M of M105 peptide, which was a concentration so high that it has no equivalent in endogenous antigen presentation on mCMV-infected cells. We have observed a similar phenomenon previously and discussed a low-avidity cross-recognition of an mCMV peptide by CD8^+^ T cells of a different specificity and a genealogy unrelated to mCMV infection [65]. Importantly, by calculating EC_50_ values only, we would have missed recognizing this contaminating population.

### 3.4. Absence of IDEs Facilitates Primarily the Expansion of High-Avidity CD8^+^ T Cells Specific for Non-IDEs

**Figure 4:**
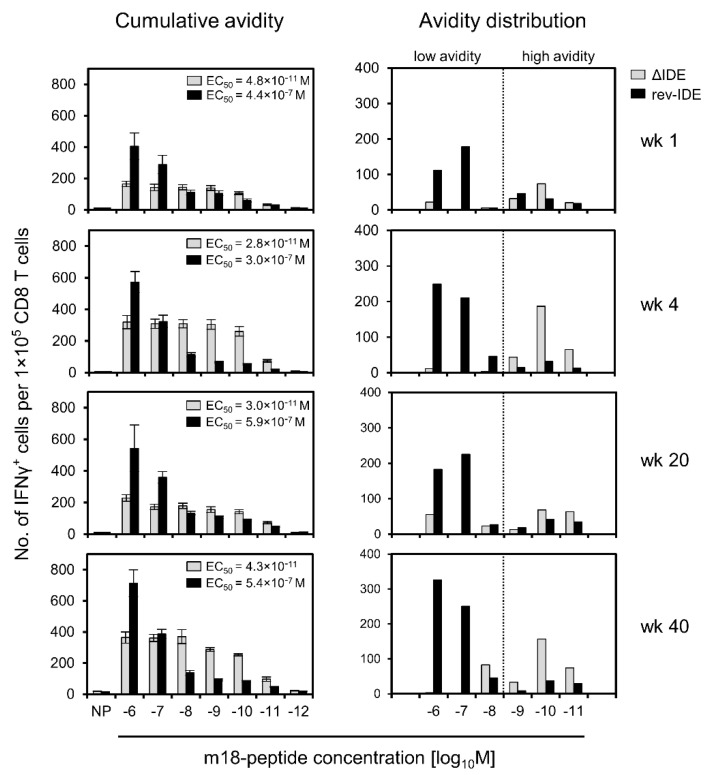
Avidity distributions of functional IFNγ^+^CD8^+^ T cells specific for non-IDE m18, depending on the absence or presence of IDEs. Immunocompetent BALB/c mice were infected via the intraplantar route with viruses ΔIDE (gray-shaded bars) or rev-IDE (black bars), respectively. At the indicated times after infection, CD8^+^ T cells specific for non-IDE m18 were quantitated based on IFNγ secretion in an ELISpot assay. Stimulation in the assay was achieved by P815 mastocytoma cells exogenously loaded with synthetic m18 peptide at the loading concentrations indicated. (Left column) Cumulative avidity plots and EC_50_ values. (Right column) Avidity distributions. For further details, see the legend to Figure 3. The dotted line operationally separates cells with non-protective avidity, equivalent to >10^−9^ M peptide loading concentration (to the left), from cells with protective avidity, equivalent to ≤10^−9^ M peptide loading concentration (to the right). To average individual variation, cells isolated from 5–10 mice, depending on cell yield, were pooled for the analysis.

Using the same approach, we determined the avidities of functional IFNγ^+^CD8^+^ T cells specific for non-IDE m18 in the time course in the absence or presence of IDEs after intraplantar infection with mCMVs ΔIDE and rev-IDE, respectively (Figure 4).

The result is utmost striking, as it clearly reveals that cells specific for non-IDE m18 that were “educated” in the presence of IDEs after infection with mCMV rev-IDE are in their vast majority of non-protective functional avidity corresponding to >10^−9^ M of m18 peptide. As a consequence, these cells will not participate in the immune control that then apparently rests on the high-avidity response to IDEs. In contrast, in the absence of IDEs after infection with mCMV ΔIDE, the avidity distribution of cells specific for non-IDE m18 shifted to the “protective zone”. Moreover, low-avidity cells were almost absent. The gain of avidity by the cell population specific for non-IDE m18 by deletion of IDEs was dramatic, as revealed by 10,000-fold decreases in the EC_50_ values (Figure 4, left column) as well as by the pronounced right-shift in the avidity distributions (Figure 4, right column) for all times of analysis. Thus, as exemplified for non-IDE m18, high-avidity cells preferentially expand in the absence of IDEs and can account for the protective activity observed in the previously published adoptive cell transfer experiments [58,59,60].

**Figure 5:**
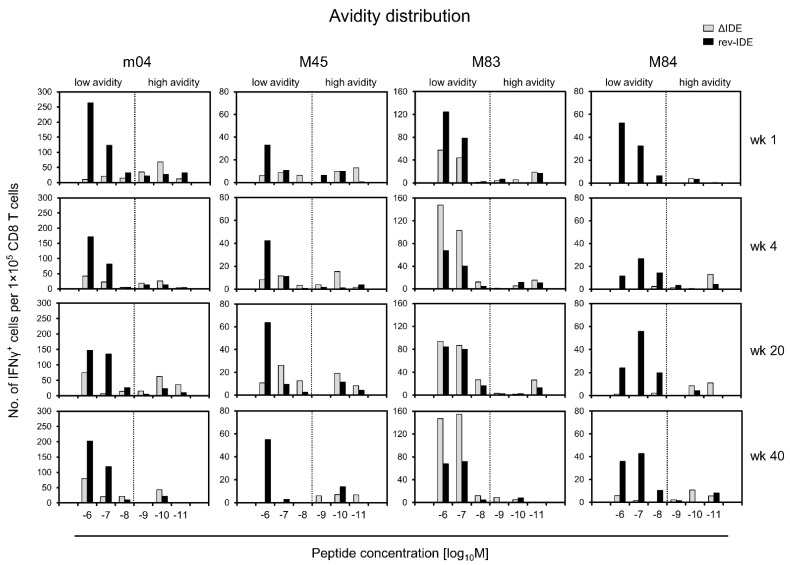
Avidity distributions of CD8^+^ T cells specific for a panel of non-IDEs dependent upon the absence or presence of non-IDEs. For more explanation, see the legends of Figure 3 and Figure 4.

As mCMV encodes a panel of additional non-IDEs for presentation in mice of the *H-2^d^* haplotype [60], we determined the avidity distributions for non-IDEs m04, M45, M83, and M84, in the absence or presence of IDEs after infection with mCMVs ΔIDE and rev-IDE, respectively (Figure 5).

At a glance, the principle shown for the chosen non-IDE paradigm m18 (Figure 4) applies also to the other non-IDEs tested, even so with distinct differences. As a common denominator, the frequencies of cells in the “protective zone” of avidities, corresponding to peptide loading concentrations of ≤10^−9^ M, are higher in the absence compared to the presence of IDEs. So, this appears to be a rule. Enrichment of low-avidity cells in the presence of IDEs, as shown for m18 (Figure 4), applies even more to peptide M84, but less and with variation over time to peptides m04 and M45. Finally, M83 represents an exception in that low-avidity cells increasingly dominated the response over time in the absence of IDEs when compared to the presence of IDEs. Importantly, these epitope-specific differences cannot be attributed to differences in the composition of the responder cell pools, because, at any time of analysis, the same polyclonal responder cell population was used for the comparison of epitopes.

## 4. Discussion

Our previous studies on the contribution of IDEs and non-IDEs to antiviral protection in different experimental in vivo settings have shown that, surprisingly, the deletion of IDEs has no noteworthy effect on the immune control [58,59,60], suggesting that the phenomenon of immunodominance has no functional relevance at all. Specifically, the resolution of acute infection in recipients of syngeneic HCT was not more efficient or faster when the virus coded for IDEs [60]. Furthermore, in adoptive cell transfer experiments, neither the absence of IDE-specific CD8^+^ T cells in donors nor the absence of IDE presentation in tissue cells of infected recipients led to a reduced control of infection. In fact, IDEs appeared to be dispensable, since the presence or absence of IDEs in both donor and recipient made no difference [59,60]. In accordance with our data on non-IDE m18 (Figure 4), this previous work had also shown that the response to known non-IDEs was not amplified in quantitative terms when IDEs were deleted. Furthermore, the absence of IDEs did not globally change the antigenic peptide repertoire, that is the antigenic peptidome, by expansion of CD8^+^ T cells specific for otherwise cryptic and thus not yet identified epitopes [59,60], with the notable exception of the M54 epitope [67]. This epitope was originally identified by ORF library screening of responder cells derived from mice at 4 weeks after syngeneic HCT and infection with mCMV ΔIDE. The expansion of these cells in the absence of IDEs was also observed in other ORF library screenings performed on day 8 after infection of immunocompetent mice [59] and at 6 months in infected HCT recipients [60], but it was less pronounced in these cases and therefore not pursued. Thus, as also shown here, precise conditions and timing matter.

We previously concluded “... that CD8^+^ T cells specific for many non-IDEs, most of which may have an individual frequency below assay detection limit, collectively mount an efficient antiviral response” [60]. Based on the data presented here, we have to revise our own previous hypothesis. Evidence is now overwhelming to conclude that the deletion of IDEs alters the response to non-IDEs qualitatively by releasing high-avidity T-cell clones specific for non-IDEs from the competition by IDEs. Consequently, non-IDEs then take on a role in antiviral protection that they do not have when IDEs are expressed. This remodeling of the response in adaptation to absence of IDEs explains why IDEs appeared to be of low importance for the immune control in our previous experiments [58,59,60].

Regarding the mechanism of competition, we puzzled over the preferential expansion of non-IDE-specific high-avidity cells in the absence of IDEs. We initially expected to find just the opposite, namely that the low-avidity response to non-IDEs ought to suffer most from competition by the strong IDEs, so that deletion of IDEs should allow their stimulation and consequent expansion. However, this assumption was made without considering the simple fact that those cells with an avidity too low for recognizing limited antigen presentation cannot take part in any competition, that is, they do not suffer from the presence of IDEs and, accordingly, cannot profit from the deletion of IDEs. This straightforward explanation must be tested in future experiments combining the deletion of IDEs with deletion of viral immune evasion genes for enhancing antigen presentation.

We are now left with the phenomenon of epitope-specific differences, in particular the deviating finding for epitope M83, showing that the low-avidity response profits from IDE deletion over time (Figure 5). At the moment, we can only speculate that the presentation of peptide M83 in the presence of immune evasion might be better than the presentation of peptide m164, for which we defined a loading peptide concentration of ≤10^−9^ M as the avidity threshold for the recognition of infected cells [65]. A shift of this threshold concentration to lower avidity in the case of M83 might explain the participation also of cells with lower avidity in the competition with IDEs.

Finally, the question remains from where low-avidity cells originate at all when antigen presentation is below the avidity threshold defined for the recognition of infected cells. We postulate that priming and later clonal expansion follow different rules. While priming can be through antigen cross-presentation by uninfected professional antigen-presenting cells circumventing viral immune evasion ([69], and references therein), expansion is likely driven by infected cells in which viral immune evasion proteins limit antigen presentation.

## 5. Conclusions

Our data provide reasonable evidence to conclude that competition by IDEs prevents the expansion of high-avidity CD8^+^ T-cell clones specific for non-IDEs. The expansion of these clones after release from competition by the deletion of IDEs explains previous data that had shown that CD8^+^ T cells specific for non-IDEs can substitute for IDE-specific cells in protection against infection with an IDE deletion mutant. From the perspective of the host, this is a mechanism for avoiding viral immune escape through antigenic drift caused by IDE mutations. In addition to this positive consequence of securing the immune control of a CMV infection, our findings may be important for the design of vaccines based on CMVs as vectors or even for vaccines based on vectors in general. Our data draw attention to the possible problem that vector-encoded IDEs impose a risk of vaccine failure by suppression of the protective CD8^+^ T-cell response directed against epitopes of the vaccine target pathogen or tumor.

## Data Availability

The data presented in this study are available on request from the corresponding author.

## Abbreviations

BAC (bacterial artificial chromosome), CMV (cytomegalovirus), cTEM (conventional T effector-memory cells), CTL (cytolytic CD8 T lymphocytes), DPEC (double-positive effector cells), EC (endothelial cells), EEC (early effector cells), GvH (Graft-versus-Host), GvHD (GvH disease), HCT (hematopoietic cell transplantation), HvG (Host-versus-Graft), hCMV (human CMV), IDE (immunodominant epitope), iTEM (inflationary T effector-memory cells), LSEC (liver-sinusoidal EC), mCMV (murine CMV), MI (memory inflation), ORF (open reading frame), PFU (plaque forming unit), SOT (solid organ transplantation), TCR (T cell receptor), TCM (T central memory cells).
